# Editorial: System Biology Methods and Tools for Integrating Omics Data

**DOI:** 10.3389/fgene.2020.563108

**Published:** 2020-11-12

**Authors:** Liang Cheng, Lei Deng, Mingxiang Teng

**Affiliations:** ^1^NHC Key Laboratory of Molecular Probe and Targeted Theranostics, Harbin Medical University, Harbin, China; ^2^College of Bioinformatics Science and Technology, Harbin Medical University, Harbin, China; ^3^School of Computer Science and Technology, Central South University, Changsha, China; ^4^Moffitt Cancer Center, Tampa, FL, United States

**Keywords:** OMICS data, data mining, machine learning, complex disease, system biology

With the rapid evolution of sequencing technologies, it becomes more and more easy for researchers to analyze the expression level of molecules or variations in the genome, transcriptome, and proteome in wet labs. These technological innovations have advanced the life science community in terms of revealing disease risk factors such as gene variations or expressions, clinical phenotypes, etc. Accompanied by technological advances, significant amounts of sequencing data have been generated in the field to then be interpreted using novel data integration methods.

To this end, it is urgent to develop methods and tools to better utilize omics datasets in disease studies. One way would be to evaluate the associations between different diseases or sub-types by analyzing omics datasets across individual laboratories. e.g., LncRNAs biomarkers, associated with clinical sub-types and the prognosis of diffuse large B-cell lymphoma, were discovered and validated by re-annotating the probes and analyzing the data of multiple microarray platforms. Another way would be to reveal potential characteristics of diseases by integrating multi-level omics data. Gene targets of complex diseases could, for example, be predicted by integrating summary data from GWAS and eQTL studies. Integration of omics data by exploring computational tools is likely to be challenging for most biologists, as most tools require a certain level of computing knowledge one the part of the users to be operated optimally. It is consequently of great import to establish automated pipelines that combine these tools. In summary, the current challenge for understanding complex disease is to mine novel and precise characterization through the fusing of multi-level omics data using system biology approaches. Here, we organized a Research Topic on “System Biology Methods and Tools for Integrating Omics Data.” In total, 22 outstanding works were presented in this thematic issue, six of which have been highlighted as follows.

- Zhao et al. integrated GWAS and eQTL of brain data to identify SNPs and genes that are related to four types of strokes (ischemic stroke, large artery stroke, cardioembolic stroke, and small vessel stroke). They explored the genetic pathogenesis based on the loci, genes, gene expression, and phenotypes. There, 38 SNPs that affect expression of 14 genes were found to be associated with stroke. Among them, one gene was found for large artery stroke, six genes for cardioembolic stroke, and eight genes for small vessel stroke. To explore the effects of environmental factors on stroke, they further identified methylation susceptibility loci associated with stroke using mQTL. A total of 31 of the 38 eQTLs were also identified as mQTLs. In a short, this study explored the genetic pathogenesis of strokes.
- Zhou et al. carried out a comprehensive analysis of single-cell genomic copy number variations (CNVs) in VHL/PBRM1-negative Clear-cell renal cell carcinoma (ccRCC). Through functional enrichment analysis, they found that the amplified genes are significantly associated with cancer-related signaling transduction pathways. Besides, receptor protein tyrosine kinase (RTK) genes also showed widespread CNVs in cancer cells. In short, their studies indicated that the genomic CNVs in RTK genes and downstream signaling transduction pathways may be involved in VHL/PBRM1-negative ccRCC pathogenesis and progression.
- Hong and Wang designed a novel method, *Frin*, for studying genome evolutionary history. Phylogenetic tree and phylogenetic network are state-of-art ways for understanding the process of biological evolution. Since each taxon in a phylogenetic tree could have more than one parent, phylogenetic trees cannot capture the complexity of evolutionary information implicit in phylogeny. Hong and Wang presented a phylogenetic network-based method *Frin* to express genome evolutionary histories. Unlike the previous methods heavily relying on the order of input data, *Frin* unified the different input orders as the same dataset for different networks.
- Han et al. explored lncRNAs of Multiple Sclerosis (MS) by integrating the RNA-seq data from multiple studies. lncRNAs were deemed as important regulatory factors in MS pathogenesis. Current research has been limited by small sample sizes or heterogeneity among various tissues. RNA-seq has become a powerful approach to quantify the abundances of lncRNA transcripts. The authors collected MS-related RNA-seq data from a variety of previous studies, and integrated the data using an expression-based meta-analysis to identify differentially expressed lncRNA between MS patients and controls in all samples and sub-groups. Results showed that a potential important function of lncRNAs may be involved in the regulation of ribonucleoproteins and TNF cytokines receptors in MS.
- Gan et al. proposed a new approach, *TriPCE*, introducing a tri-clustering strategy to integrative pan-cancer epigenomic analysis. *TriPCE* can identify coherent patterns of various epigenetic modifications across different cancer types. To validate its capability, they applied *TriPCE* to analyze six important epigenetic marks among seven cancer types and identified significant cross-cancer epigenetic similarities. The results highlighted specific epigenetic patterns among the investigated cancers. The functional gene analysis further demonstrated strong relevance of studied gene sets with cancer development and revealed a consistent risk tendency among these investigated cancer types.
- Zeng et al. developed a hybrid deep neural network framework *4mcDeep-CBI*, aiming to identify 4mC sites. Preliminary extracted features were fed to the Convolutional Neural Network (CNN) and Bidirectional Long Short Term Memory network (BLSTM) to generate advanced features. Taking the advanced features as input, they designed an integrated algorithm to improve feature representation. Experimental results on a large new dataset showed that *4mcDeep-CBI* could achieve generally better performances when identifying 4mC sites compared to other state-of-art predictors.

Each study in the special issue was peer reviewed by two or three external reviewers. We would like to thank all the authors for contributing their work to our hot thematic issue and all the reviewers for their time and efforts. Finally, we would like to thank the Chief Editor and Editorial Office of Frontiers in Genetics for their support during the whole processes.

## Author Contributions

LC, LD, and MT conducted this topic issue and wrote the manuscript. All authors contributed to the article and approved the submitted version.

## Conflict of Interest

The authors declare that the research was conducted in the absence of any commercial or financial relationships that could be construed as a potential conflict of interest.

